# Acceptance of Enhanced Robotic Assistance Systems in People With Amyotrophic Lateral Sclerosis–Associated Motor Impairment: Observational Online Study

**DOI:** 10.2196/18972

**Published:** 2021-12-06

**Authors:** André Maier, Cornelia Eicher, Joern Kiselev, Robert Klebbe, Marius Greuèl, Dagmar Kettemann, Marcel Gaudlitz, Bertram Walter, Ursula Oleimeulen, Christoph Münch, Thomas Meyer, Susanne Spittel

**Affiliations:** 1 Center for ALS and other Motor Neuron Disorders Charité – Universitätsmedizin Berlin Corporate Member of Freie Universität Berlin, Humboldt-Universität zu Berlin, and Berlin Institute of Health, Germany Berlin Germany; 2 Working Group on Aging & Technology Charité – Universitätsmedizin Berlin Corporate Member of Freie Universität Berlin, Humboldt-Universität zu Berlin, and Berlin Institute of Health, Germany Berlin Germany; 3 Department for Anesthesiology and Intensive Care Medicine Campus Charité Mitte and Campus Virchow-Klinikum Charité – Universitätsmedizin Berlin Corporate Member of Freie Universität Berlin, Humboldt-Universität zu Berlin, and Berlin Institute of Health Berlin Germany; 4 Pflegewerk Berlin GmbH Berlin Germany; 5 Ambulanzpartner Soziotechnologie APST GmbH Berlin Germany

**Keywords:** amyotrophic lateral sclerosis, assistive robotics, technology commitment, robotic arm assistance

## Abstract

**Background:**

Amyotrophic lateral sclerosis (ALS) is a fatal neurodegenerative disease characterized by a progressive paresis of the extremities and the loss of manual functioning. Due to the severe functional impairment that the disease entails, ALS requires the provision of comprehensive nursing care and a complex set of assistive technology devices. To relieve caregivers and promote autonomy of people with ALS, robotic assistance systems are being developed. This trial aims to evaluate the acceptance of technology, in general, and of robotic arm assistance among people with ALS in order to lay the groundwork for the development of a semiautomatic robotic arm that can be controlled by humans via a multimodal user interface and that will allow users to handle objects and attend to their own bodies.

**Objective:**

The aim of this study was to perform a systematic analysis of technology commitment and acceptance of robotic assistance systems from the perspective of physically limited people living with ALS.

**Methods:**

The investigation was conducted as a study of a prospective cohort. Participants were only included if they had received a medical diagnosis of ALS. Data collection took place via an online questionnaire on the Ambulanzpartner Soziotechnologie internet platform. Technological commitment was measured using the Neyer short scale. Furthermore, a multidimensional questionnaire was specially developed to analyze participant acceptance of robotic arm assistance: the Acceptance Measure of Robotic Arm Assistance (AMRAA). This questionnaire was accompanied by a video introducing the robot arm. ALS severity was ascertained using the ALS Functional Rating Scale–Extended (ALSFRS-EX).

**Results:**

A total of 268 people with ALS participated in the survey. Two-thirds of the participants were male. The overall mean ALS severity score was 42.9 (SD 11.7) points out of 60 on the ALSFRS-EX, with the most relevant restrictions on arms and legs (<60% of normal functioning). Technological commitment ranked high, with the top third scoring 47.2 points out of 60. Younger participants and males showed significantly higher values. The AMRAA score was, again, significantly higher among younger participants. However, the gender difference within the overall cohort was not significant. The more limited the arm functioning of participants according to the ALSFRS-EX subscale, the higher the acceptance rate of robotic assistance. This relationship proved significant.

**Conclusions:**

People with ALS display high technological commitment and feel positive about using technological assistance systems. In our study, younger participants were more open to technology use, in general, and robotic assistance, in particular. Self-appraisal of technology acceptance, competence, and control conviction were generally higher among men. However, any presumed gender difference vanished when users were asked to rate the anticipated usefulness of the technology, in particular the robotic arm. The acceptance was also reflected in users’ increased willingness to use a robotic arm as the functionality of their own arms decreased. From the perspective of people with ALS, robotic assistance systems are critical to promoting individual autonomy. Another key consideration in the development of future assistive technologies should be the reduction of caregiver burden.

**Trial Registration:**

German Clinical Trials Register DRKS00012803; https://tinyurl.com/w9yzduhd

## Introduction

Amyotrophic lateral sclerosis (ALS) is a fatal neurodegenerative disease characterized by a progressive paresis of the extremities, loss of manual functioning, and a high degree of need for long-term care within 2 to 4 years. Cognitive functions are mostly unaffected, but people with ALS typically develop dysphagia and dysarthria as tongue and pharyngeal muscles weaken. Prevalence peaks in the seventh decade of life [1]. Because of the severe functional impairment it causes, ALS treatment requires the provision of comprehensive nursing care and a complex set of assistive technology devices (ATDs) [2]. The most common ATDs are home modifications, daily living devices, orthoses, transfer devices, augmentative and alternative communication devices, and mobility devices, such as electric and manual wheelchairs [2-4].

Robotic assistance systems for physically impaired people, like robotic arms, have recently been introduced into the field of medical devices [5]. These systems are designed to compensate for motor limitations of the hands and arms, particularly with regard to fine motor skills and grabbing. Advanced robotic assistance systems enable users to handle objects and attend to their own bodies. There are manifold ways to control robotic devices. If it is no longer feasible for a person to use a joystick or a point-and-click cursor, eye control and speech amplification are other options. Even brain-computer interfaces have been successfully evaluated in research environments, but they can only be implemented under certain conditions [6]. For applications such as drinking and feeding, autonomous [7] or semiautonomous [8,9] approaches are currently under evaluation.

Automatized and intelligent robotic assistance systems are designed not only to promote individual autonomy but also to relieve caregiver burden. The burden on caregivers is likely to increase in parallel to the severity of the disease, and is exacerbated by the general diminishment of physical functioning of the person concerned, which, in turn, can elevate caregiver stress levels [10]. In the later stages of ALS, the demands for assistance and treatment measures increase and become of greater importance. In particular, the repeated performance of small and comforting actions (eg, minimal repositioning of extremities, scratching, itching, wiping off saliva in cases of sialorrhea, and correcting head positioning during the use of eye-controlled communication devices) can lead to stress and demoralization among caregivers and nursing professionals.

Since assistive robotic technology has become a subject of academic research, there has been a debate about its acceptance, especially among older adults [11]. This discussion often assumes that there are basically two realms in which assistive robots can be useful: the physical and the social. However, robotic applications do not have to be limited to these categories and can intervene in both. While even skeptics concede that robots are better able to perform certain standardized tasks than human caretakers, introducing robot-human interaction in a caregiving context may still be considered controversial. Physically assistive robots, on the other hand, are complex tools that can be widely implemented, and as with other technologies that are developed incrementally, it is more likely that they will be accepted.

The aim of this study was to investigate the acceptance of robotic assistance systems among people with ALS with regard to their physical impairment and willingness to accept technology-based care. As of today, no such structured data are available on these forms of ALS treatment.

## Methods

### Study Design

This observational study employed a cross-sectional descriptive design to perform a quantitative requirements analysis for the research and development project ROBINA (robot-supported services for individual and resource-oriented care of patients with ALS) [4,12]. It complies with the Checklist for Reporting Results of Internet E-Surveys (CHERRIES) guidelines [13]. The data were compiled through a closed online survey comprised of four different parts that addressed the research question and was tailored to the target group. Two validated and standardized questionnaires pertaining to motor functioning and technology commitment were followed by a video of the research robot (Multimedia Appendix 1) and the newly developed Acceptance Measure of Robotic Arm Assistance (AMRAA) with reference to that video (Multimedia Appendix 2).

### Setting and Recruitment

To reach a broad convenience sample of participants with ALS, we created an online survey using an open-source web application designed by LimeSurvey [14]. The application was embedded into the protected internet platform of Ambulanzpartner Soziotechnologie (APST) [15]. The APST platform provides users with access to specialized therapists and coordinators that focus on case management, and has a tailored digital management platform with tools for self-assessment, medical services, therapy, and assistive devices [3]. This digital and internet-supported case management network has existed since 2011 and, at the time of our survey, it coordinated the care requirements of more than 3700 people who had been diagnosed with ALS according to the revised El Escorial World Federation of Neurology criteria [16]. Those people and their caregivers were granted access to the APST platform through private individual accounts. In joining the network, participants consented to possible future contact from scientific institutions as approved by the Berlin Institutional Review Board and Data Security.

### Participants

Requests to participate in the survey were submitted via email to approximately 2600 registered members of the APST platform. To qualify for our survey, participants needed to have a confirmed medical diagnosis of ALS following the revised El Escorial World Federation of Neurology criteria [16]. After registering with APST, participants provided medical documentation containing their diagnosis, including a physician’s letter, which was subsequently entered into the database by an experienced case and data manager.

### Variables and Data Sources

#### Brief Measure of Technology Commitment

Technology commitment gauges individual willingness to use technology via three distinct domains: technology acceptance, technology competence conviction, and technology control conviction [17]. Neyer et al developed a model for measuring and scaling technology commitment via these three domains. The model is premised on 12 statements upon which respondents agree or disagree on a scale from 1 (fully agree) to 5 (fully disagree). Each domain correlates to four statements, and the results can be analyzed individually or as a whole; the total score ranges from 12 to 60, with a high value corresponding to a higher general commitment to technology. The technological competence conviction numbers must be re-encoded when calculating the final score, as its statements are phrased in negative terms.

#### ALS Functional Rating Scale–Extended

We evaluated the functional impairment of participants using the ALS Functional Rating Scale–Extended (ALSFRS-EX), which was developed in an online community [18] and subsequently validated in German [19]. We found that the long-standing predecessor of this instrument, the ALS Functional Rating Scale–Revised (ALSFRS-R), produced comparable results to in-clinic evaluation, even though our testing was performed online [20]. The extended version includes three additional questions, which enhances the sensitivity of the score by better reflecting the deterioration of physical functioning that occurs in advanced stages of ALS. In particular, by inquiring about the operability of buttons, the ALSFRS-EX prioritizes manual functioning and focuses on the motor restrictions that are relevant to this project. In addition to assessing fine motor functions, each individual score assesses gross motor functions of the upper and lower extremities, bulbar functions, and breathing abilities. The survey is comprised of 15 short, clear questions with responses given on a 5-point scale ranging from 0 (total loss) to 4 (fully preserved). Hence, the total score for the scale ranges from 0 to 60 points, with fewer points representing more severe symptoms. The loss of ALSFRS points per month, or delta ALSFRS, indicates the rate of deterioration and predicts survival [14].

#### Video of the Enhanced Research Robot

Our research program aims to develop robotic assistance and resource-oriented care for individual people with ALS. Assistive robots should not only be controllable by the patient through an interface, but they should also be able to perform minimal comforting actions with partial autonomy. The most advanced robotic arm in Germany—and perhaps anywhere in the world—is our technological starting point. The Franka Emika Panda is an industrial robot designed with a sense of touch and equipped with sensors [21]. The robot is intended to work with humans, and, in the future, it should be able to autonomously perform simple tasks, likely by integrating object recognition. To illustrate a robotic arm and the robot’s potential, participants were shown a short video highlighting several features of the arm (see Multimedia Appendix 1 for the video). At the time of our survey, the most recent state of development was not available, but developers were training the robot to scratch participants’ skin with a small brush and have it detect and reach for objects.

#### Acceptance Measure of Robotic Arm Assistance

We developed a new measurement tool, the AMRAA, to evaluate how willing participants would be to accept the assistance of a robotic arm. The items and domains of this tool were developed by a group of experts from the fields of ALS research, ALS care, and aging and technology. This instrument consists of 10 statements upon which respondents agree or disagree on a 6-point Likert scale ranging from 0 (fully disagree) to 5 (fully agree). The items are merged into three domains: experience with robotic assistance (2 items), current need of robotic arm assistance (4 items), and future usage of robotic arm assistance (4 items). A maximum total score of 50 points can be achieved if the individual agrees to each statement to the greatest possible extent. A version of this scale translated into English is in Multimedia Appendix 2.

The instrument went through a pretest process with people with ALS, after which the statements were strengthened. No complete validation process was carried out in advance of its use; however, rotated component analysis confirmed a strong loading (λ>0.6) of the items in the three domains of the questionnaire, with a balanced cross-loading of two items between current need and future usage domains (Multimedia Appendix 3).

### Data Analysis

Data were analyzed with SPSS Statistics for Windows (version 25; IBM Corp). Results were expressed as means and SDs if normally distributed, and medians and IQRs if numerical data were visualized or if distribution was non-Gaussian. Correlational analysis was performed with Spearman ρ because of the ordinal nature of the scales. For group differences of nonparametric data, the Mann-Whitney *U* test was performed for two independent samples, and the Kruskal-Wallis one-way analysis of variance was performed for three independent samples. Factor analysis was conducted using the iterated principal factor method with varimax rotation. A *P* value of <.05 (two-tailed) was considered significant.

### Protocol Approvals and Registrations

People who participate in the APST network agree to take part in scientific surveys and trials. Informed consent forms were obtained from all participants. Furthermore, the online survey and study protocol have been approved by the Medical Ethics Committee of the Charité – University Hospital Berlin, Germany, as a part of the requirements analysis for the ROBINA project with a mixed methods approach (approval No. EA1/121/17). The trial has been registered at the German Clinical Trials Register (DRKS00012803) and with the World Health Organization International Clinical Trials Registry Platform.

## Results

### Overview

A total of 268 participants, 10.1% of all persons queried across 16 ALS centers in Germany, took part in the online survey. Of the total number of participants, 53.4% (n=143) were patients of the ALS outpatient department at Charité – University Hospital Berlin.

The mean age of all participants at the time of response was 60 (SD 10.6) years (range 33-87); participants had a median disease duration of 27 (IQR 41) months (range 2-227). There was a comparatively high percentage of long-term survivors in our trial. Out of all participants, 22.0% (n=59) had a disease duration of 5 years or more, which is why the cohort showed a relatively slow median course of the disease on average. Table 1 summarizes the baseline population characteristics of all participants.

**Table 1:** Participant demographics and baseline disease characteristics.

Characteristic			Value (N=268)
Age (years), mean (SD)			60 (10.6)
**Gender, n (%)**			
	Female	88 (32.8)	
	Male	180 (67.2)	
ALSFRS-EX^a^ baseline score, mean (SD)			42.9 (11.7)
Delta ALSFRS-EX^b^, median (IQR, range)			0.56 (0.81, 0.01-4.5)
Duration of disease (months), median (IQR, range)			27.0 (41, 2-213)

^a^ALSFRS-EX: Amyotrophic Lateral Sclerosis Functional Rating Scale–Extended.

^b^Loss of ALSFRS-EX points per month.

### Main Results

#### Brief Measure of Technology Commitment

**Figure 1:**
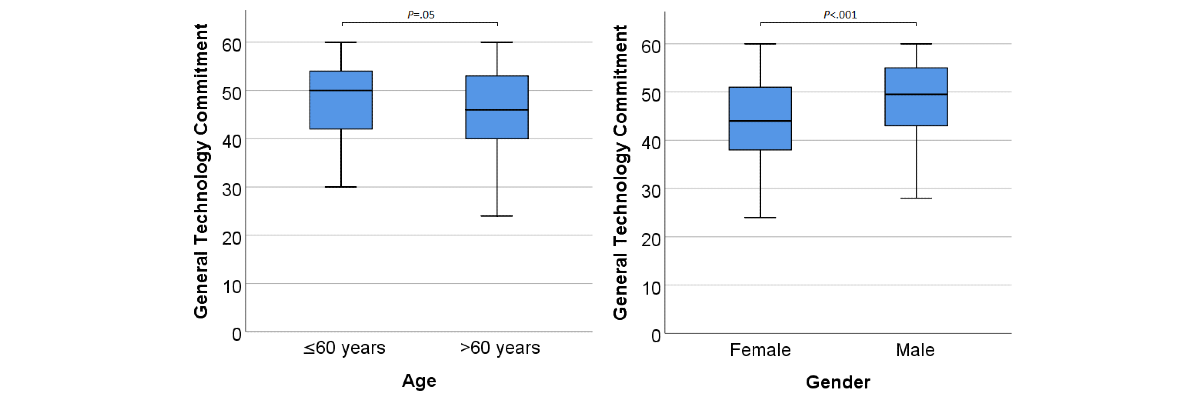
General technology commitment as a function of age and gender. The horizontal lines in the blue boxes represent the medians. Whiskers indicate minimum and maximum. *P* values were based on the Mann-Whitney *U* test.

The general commitment to technology use was high across all age groups (mean 47.2, SD 8.2, out of 60 points), but was significantly higher among participants under 60 years of age (Figure 1). Males showed a significantly higher technology commitment when compared to females (median 49.5 vs 44 points, respectively; *P*<.001; Figure 1).

**Figure 2:**
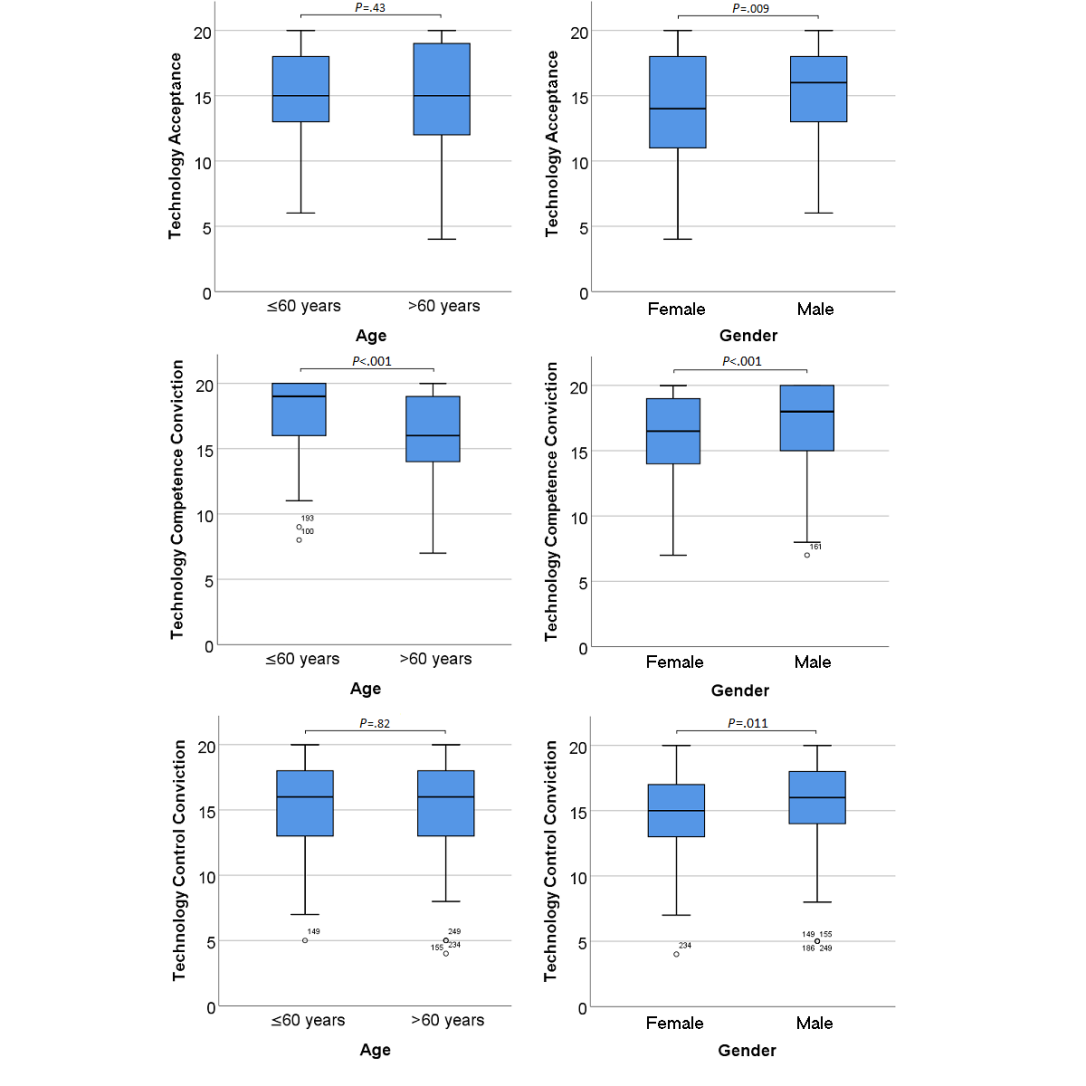
Single domains of technology commitment as a function of age and gender. The horizontal lines in the blue boxes represent the medians. Whiskers indicate minimum and maximum. Circles on plots represent outliers outside the 1st or 3rd quartiles ±1.5 × IQR. *P* values were based on the Mann-Whitney *U* test.

A gender difference was also evident in the three domains of technology commitment (Figure 2). The self-assessment showed a significantly higher technology competence conviction among younger participants (age ≤60 years; *P*<.001; Figure 2).

There was no difference between age groups in terms of technology acceptance and technology control conviction. Within our cohort, the increasingly restricted arm functioning, as measured by the ALSFRS-EX subscale, had no measurable effect on general technology use commitment or its domains.

#### ALS Functional Rating Scale–Extended

Respondents had a mean ALSFRS-EX score of 42.9 (SD 11.7) at the time of the survey, with a median loss since disease onset of 0.56 (IQR 0.81) points per month on average. Within our population, people were most severely impaired in arm and leg functioning. As depicted in Table 2, upper and lower limbs were functioning, on average, at less than 60% of normal rates. Bulbar and respiratory functions were less affected. This distribution can be explained if we assume that the majority of affected participants initially had symptoms in their extremities, which is referred to as spinal onset, and which occurs in about 80% of German cohorts [22].

**Table 2:** Functional impairment of participants according to the ALSFRS-EX and its domains.

ALSFRS-EX^a^ domain	Maximum reachable points, n	Achieved points, mean (SD)	Relative function, %
Swallowing, speech, and facial expression	16	12.3 (4.0)	76.9
Upper limbs: finger and arm function	16	9.6 (5.0)	58.7
Lower limbs: walking and leg function	16	9.5 (4.9)	59.4
Dyspnea and breathing: respiratory function	12	10.5 (2.2)	83.7
All domains	60	42.9 (11.7)	71.4

^a^ALSFRS-EX: Amyotrophic Lateral Sclerosis Functional Rating Scale–Extended.

#### Acceptance Measure of Robotic Arm Assistance

**Figure 3:**
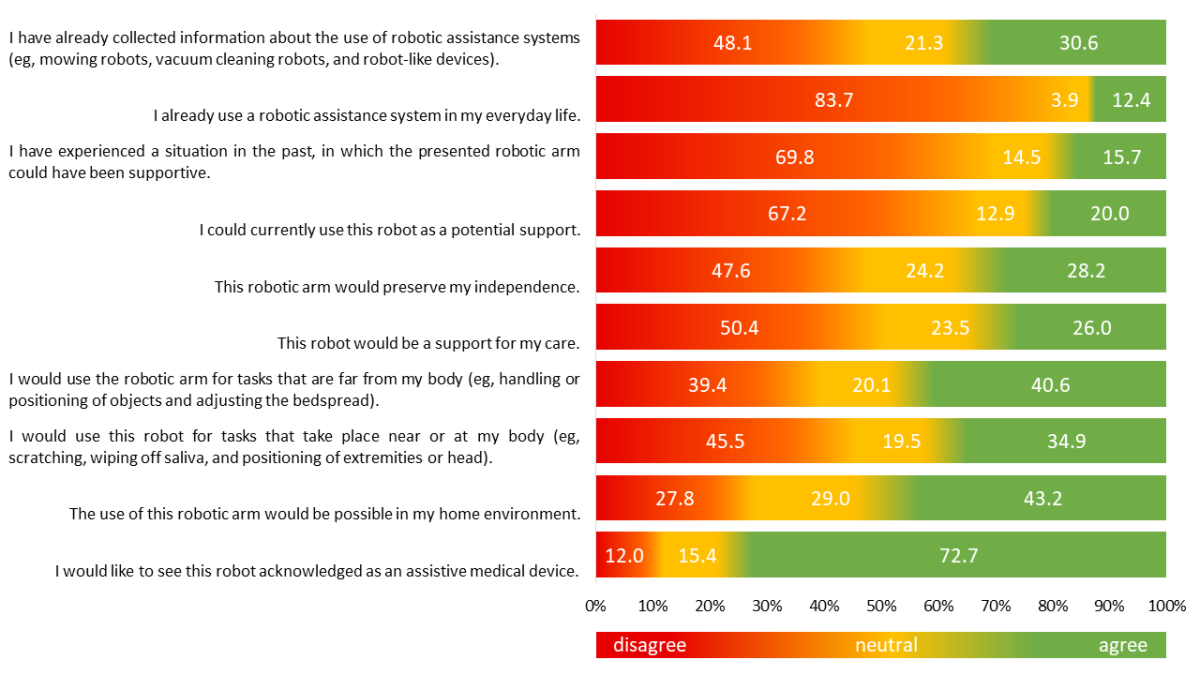
Acceptance Measure of Robotic Arm Assistance (AMRAA) ratings of statements about robotics and robotic arm assistance. Percentages of the ratings at each level (disagree = 0-1, neutral = 2-3, and agree = 4-5) for each statement are shown on the bars.

Before participants were asked to rate statements about robotics and robotic arm assistance on the newly developed AMRAA, a video about a robotic arm was presented. Of the participants who evaluated the particular statements, 30.6% (79/258) reported they had already gathered information on robotic assistance systems. Additionally, 12.4% (32/258) were already using robotic assistance systems in their daily lives (eg, robotic lawn mower and robotic vacuum cleaner). A total of 19.9% (51/256) of respondents stated that they wanted robotic assistance for their daily care. With regard to the statement that a robot arm would support their independence, 28.2% (70/248) agreed. Moreover, 40.6% (99/244) of participants could imagine using robotic assistance systems for actions performed far from their bodies (ie, outbound activities, such as passing objects), and 35.0% (86/246) could imagine using a robotic device to attend to their own body (ie, inbound activities, such as wiping off saliva and scratching). The vast majority of participants (175/241, 72.6%) felt that robotic assistance systems should be established as prescribed medical devices, as shown in Figure 3.

**Figure 4:**
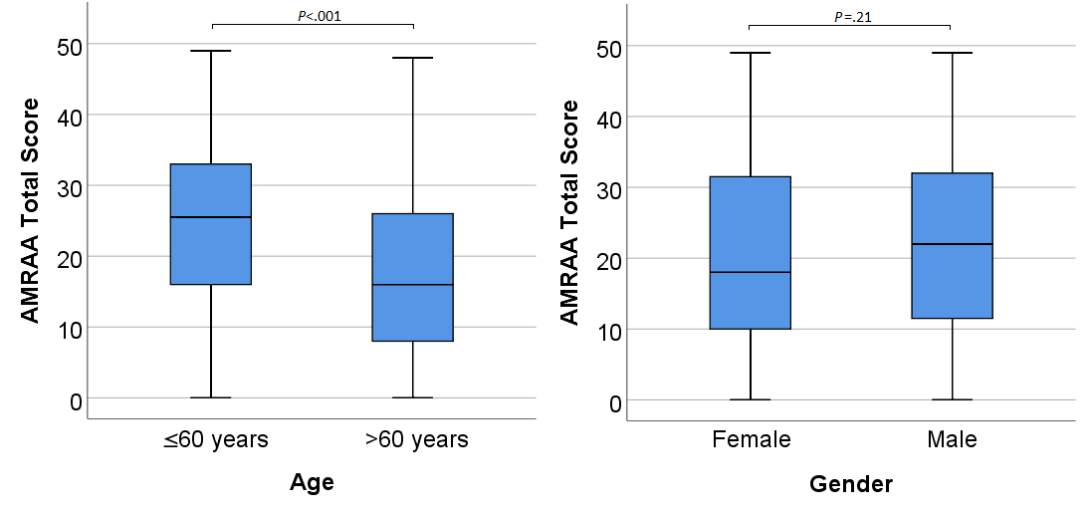
Acceptance Measure of Robotic Arm Assistance (AMRAA) scores as a function of age and gender. The horizontal lines in the blue boxes represent the medians. Whiskers indicate minimum and maximum. *P* values were based on the Mann-Whitney *U* test.

Younger participants showed a significantly higher willingness to use robotic arm assistance compared to older participants (median 25.5, IQR 17.25, vs median 16, IQR 18.5, respectively; *P*<.001; Figure 4). Higher acceptance among younger participants was also present across all domains of the questionnaire (Table 3; Multimedia Appendix 4).

There was no significant gender difference in the general acceptance of a robotic arm, although men tended to be more receptive. Interestingly, in the domain “experience with robotic assistance,” the gender difference proved significant (*P*=.03; Table 3; Multimedia Appendix 4).

**Table 3:** AMRAA scores by domain for age and gender groups.

AMRAA^a^ domain	Age (years), median (IQR)		*P* value^b^	Gender, median (IQR)			*P* value^b^
	≤60	>60		Female	Male		
Current need of robotic assistance (4 items)	8 (10)	3 (9)	<.001	4 (10)	6 (10)	.27	
Future usage of robotic assistance (4 items)	15 (9.5)	10 (10.5)	<.001	11 (12)	13 (10)	.24	
Experience with robotic assistance (2 items)	3 (5)	2 (4)	.003	1 (4)	2 (5)	.03	
All domains	25.5 (17.25)	16 (18.5)	<.001	18 (21.75)	22 (21)	.21	

^a^AMRAA: Acceptance Measure of Robotic Arm Assistance.

^b^*P* values were based on the Mann-Whitney *U* test.

Based on the ALSFRS-EX subscale for arm functioning, we categorized participants into three groups: “slightly to not restricted” (8-12 out of 12 points), “moderately restricted” (4-7 out of 12 points), and “highly restricted” (0-3 out of 12 points). Within the respective groups, we found that the more limited the arm functioning, the higher the acceptance of robotic assistance. This relationship proved significant (Table 4 and Multimedia Appendix 5). This relationship was most evident in the domains of “current need of robotic assistance” and “future usage of robotic assistance.” A decrease in arm functioning was also moderately correlated [23] with the AMRAA score (*r*=0.32, *P*=.01; Table 4).

**Table 4:** Group differences between AMRAA total and domain scores for the three ALSFRS-EX arm functioning subscale groups, and correlations between AMRAA scores and arm functioning.

AMRAA^a^ domain	AMRAA scores for ALSFRS-EX^b^ arm functioning subscale groups, median (IQR)				*P* value^c^		Correlation between AMRAA score and arm function, *r*		*P* value^d^
	Slightly to not restricted	Moderately restricted	Highly restricted						
Current need of robotic assistance	4 (8)	7 (11.25)	10 (11)	<.001		0.38		.01	
Future usage of robotic assistance	10 (10.25)	14 (10.25)	15 (9)	.03		0.22		.01	
Experience with robotic assistance	2 (4)	2 (5)	3 (7)	.23		0.08		.23	
All domains	17 (17.25)	22 (22.25)	32 (20)	<.001		0.32		.01	

^a^AMRAA: Acceptance Measure of Robotic Arm Assistance.

^b^ALSFRS-EX: Amyotrophic Lateral Sclerosis Functional Rating Scale–Extended.

^c^*P* values for group differences were based on the Kruskal-Wallis test.

^d^*P* values for correlation analysis were based on Spearman ρ.

## Discussion

### Principal Findings

The aim of this study was to evaluate the principal needs and conditions for the care and maintenance of people with ALS through robotic arm assistance. By using the APST network, we were able to recruit a high number of participants from ALS centers all over Germany to take part in this online survey. The study was part of the requirements analysis for the development of a semiautonomous robotic arm for people with ALS.

Participants self-assessed a high degree of technology commitment, regardless of motor restriction. Males, as compared to females, achieved significantly higher values in all three domains and higher composite scores. Age was a factor in how participants judged their own technological competence. Younger participants credited themselves with greater competence in dealing with new technologies. Interestingly, self-assessment rates via technological acceptance and the belief in being able to control technology were not significantly lower among older participants compared to younger participants.

After presenting a video of an assistive robotic arm prototype, we gave participants a newly developed questionnaire on this particular robotic arm. The results were comparable to the general outcome of technology commitment, but a gender-specific difference was much less obvious. There was clear evidence that younger participants, as compared to older participants, would prefer to use a robotic arm. This result is consistent with other studies on technology acceptance and is attributed to the fact that younger people are more familiar and experienced with new technologies [24].

In the context of moderating variables, the degree of physical limitation was a key factor in technology acceptance and intention to use technology. We also observed that the degree to which manual and arm functioning were impaired had a considerable effect on the fundamental attitudes toward the robotic arm. The more advanced the functional limitation of their arms, the more the participants could imagine using robotic assistance.

Interactions with the robotic arm, which attends to one’s own body compared to the application leading away from the body, were positively evaluated by slightly fewer participants, but this difference was negligible.

Interestingly, gender played no relevant role in the demand for, or acceptance of, a robotic arm. This is of interest because technological self-efficacy is lower among women, which may have an impact on perceived usefulness. In addition, the literature suggests that women’s acceptance of technology is often related to ease of use rather than the usefulness of a particular technology for a particular purpose [25]. Although in our study the use of a robotic assistive device was only shown on video, it can be assumed that this demonstration made the perceived utility more understandable. In this context, Flandorfer refers to how moderating factors, such as previous experience with the technology, can have a mediating effect, especially in counteracting age and gender differences [24]. With regard to acceptance and use patterns, studies also show the importance of positive experiences in dealing with innovative technologies, especially for user groups that are characterized by low self-efficacy and greater reluctance to use technology [24]. In addition to physical limitations, however, some people with ALS show cognitive deficits and affective disorders [26]. Since such mental illnesses can reduce the acceptance of assistive robotics, their use should be adapted to meet individual needs [27,28]. Participants also recognized the fact that the robotic arm would support caregivers and assessed the benefit to them as comparable to their own benefit.

Given the socioeconomic and psychosocial focus of ALS treatment and care, assistive technologies represent a win-win-win solution: they not only ease difficulties for functionally impaired people, but their production also propels the economy and their use addresses challenges presented by the shortage of working nurses in aging populations. Optimal and targeted handling of assistive robotic arms should minimize obstacles to implementation, and the use of such systems will improve the care and autonomy of people with ALS. This is underlined by the fact that the possibility of using robotic arms as assistive devices was supported by an overwhelming majority of respondents.

### Limitations

The crucial limitation of questionnaire-based surveys is that these instruments restrict conclusions to certain concepts framed by terminology. The newly developed AMRAA has not yet been validated and may still be further refined. A certain acquiescence tendency caused by the statements and even the aforementioned video, despite the ambition of maintaining neutrality, may lead to response bias. However, the insights that this instrument allows for are valuable for the establishment of robotics as an assistive framework and it has the potential to be used in further studies on the acceptance of ATDs.

Further limitations to our study were that we reached out to participants via email, and we conducted our survey online. It must, therefore, be assumed that those who participated had a higher technological affinity or at least good access to technology. Members of our population were treated at specialized centers in a technologically advanced country; therefore, our findings may not be applicable to other populations.

People with impaired arms, such as people with ALS, may not only acknowledge the benefits of a robotic arm, but they may also have more experience with assistive technologies and, therefore, be more motivated to use them [29]. Lastly, our trial did not focus on key sociodemographic factors, such as socioeconomic status, family and care situation, or cultural background.

### Conclusions

The robotic arm supports people with limited functioning in performing elementary manual actions autonomously, such as gripping and handling, with the aid of a device. The use of assistive robotics can increase individual independence with regard to daily activities and motor self-determination. This study identifies the existing demand for assistive robotics and the relationship between this demand and functional limitations. Establishing the general and specific technological commitments of people with ALS is an important precondition for integrating the provision of a robotic arm into an individual, participatory, and autonomy-oriented understanding of medical aid in ALS treatment. Future studies should investigate how these assistive technologies improve the everyday function of grasping and, thus, the quality of life of people with ALS.

## Appendix

Multimedia Appendix 1 Video of the robotic arm.

Multimedia Appendix 2 Acceptance Measure of Robotic Arm Assistance (AMRAA).

Multimedia Appendix 3 Rotated component matrix of the Acceptance Measure of Robotic Arm Assistance (AMRAA).

Multimedia Appendix 4 Acceptance Measure of Robotic Arm Assistance (AMRAA) scores for various domains as a function of age and gender. The horizontal lines in the blue boxes represent the medians. Whiskers indicate minimum and maximum. <italic>P</italic> values were based on the Mann-Whitney <italic>>U</italic> test.

Multimedia Appendix 5 Group differences between the total AMRAA score and three AMRAA domain scores for the three ALSFRS-EX arm functioning subscale groups: "slightly to not restricted," "moderately restricted," and "highly restricted." <italic>P</italic> values for group differences were based on the Kruskal-Wallis test. ALSFRS-EX: Amyotrophic Lateral Sclerosis Functional Rating Scale–Extended; AMRAA: Acceptance Measure of Robotic Arm Assistance.

## Abbreviations

ALS: amyotrophic lateral sclerosis
ALSFRS: Amyotrophic Lateral Sclerosis Functional Rating Scale
ALSFRS-EX: Amyotrophic Lateral Sclerosis Functional Rating Scale–Extended
ALSFRS-R: Amyotrophic Lateral Sclerosis Functional Rating Scale–Revised
AMRAA: Acceptance Measure of Robotic Arm Assistance
APST: Ambulanzpartner Soziotechnologie
ATD: assistive technology device
CHERRIES: Checklist for Reporting Results of Internet E-Surveys
ROBINA: robot-supported services for individual and resource-oriented care of patients with amyotrophic lateral sclerosis
