# Supplementary material for: Acceptance of Enhanced Robotic Assistance Systems in People With Amyotrophic Lateral Sclerosis–Associated Motor Impairment: Observational Online Study
Source: JMIR Rehabil Assist Technol. 2021 Dec 6;8(4):e18972. doi: 10.2196/18972 (PMC8691409; doi:10.2196/18972)
Supplement: Multimedia Appendix 2 [file rehab_v8i4e18972_app2.pdf]

Acceptance Measure of Robotic Arm Assistance (AMRAA)

The aim of this questionnaire is to investigate the acceptance of robotic arm assistance by functionally impaired persons. Please watch the following video (duration: 1 min.) and assess the 10 statements from your position.

https://www.youtube.com/watch?v=EtguZCu5blQ#action=share

Please evaluate the statements spontaneously and use the lines for comments and notes.

**1**	***experience with robotic assistance***		0	1	2	3	4	5		Prefer not to say:
1.1	I have already collected information about the use of robotic assistance systems (eg, mowing robots, vacuum cleaning robots, and robot-like devices).	Fully disagree	☐	☐	☐	☐	☐	☐	Fully agree	☐
	Comment:		______							
1.2	I already use a robotic assistance system in my everyday life.	Fully disagree	☐	☐	☐	☐	☐	☐	Fully agree	☐
	Comment:		______							

**2**	***current need of robotic assistance***									
2.1	I can remember a situation where this robotic arm would have been helpful for me.	Fully disagree	☐	☐	☐	☐	☐	☐	Fully agree	☐
	Comment:		______							
2.2	I could currently use this robot as a potential support.	Fully disagree	☐	☐	☐	☐	☐	☐	Fully agree	☐
	Comment:		______							
2.3	This robotic arm would preserve my independence.	Fully disagree	☐	☐	☐	☐	☐	☐	Fully agree	☐
	Comment:		______							
2.4	This robot would be a support for my caregivers.	Fully disagree	☐	☐	☐	☐	☐	☐	Fully agree	☐
	Comment:		______							

**3**	***future usage of robotic assistance***									
3.1	I would use the robotic arm for tasks that are far from my body (eg, handling or positioning of objects and adjusting the bedspread).	Fully disagree	☐	☐	☐	☐	☐	☐	Fully agree	☐
	Comment:		______							

3.2	I would use this robot for tasks that take place near or at my body (eg, scratching, wiping off saliva, or positioning of extremities or head).	Fully disagree	☐	☐	☐	☐	☐	☐	Fully agree	☐
	Comment:									
3.3	The use of this robotic arm would be possible in my house.	Fully disagree	☐	☐	☐	☐	☐	☐	Fully agree	☐
	Comment:									
3.4	I would like to see this robot acknowledged as an assistive medical device.	Fully disagree	☐	☐	☐	☐	☐	☐	Fully agree	☐
	Comment:									
