# Supplementary material for: Acceptance of Enhanced Robotic Assistance Systems in People With Amyotrophic Lateral Sclerosis–Associated Motor Impairment: Observational Online Study
Source: JMIR Rehabil Assist Technol. 2021 Dec 6;8(4):e18972. doi: 10.2196/18972 (PMC8691409; doi:10.2196/18972)
Supplement: Multimedia Appendix 3 [file rehab_v8i4e18972_app3.pdf]

**Rotated Component Matrix[a]**

| | Component | | |
|---|---|---|---|
| | 1 | 2 | 3 |
| I have already collected information about the use of robotic assistance systems (eg, mowing robots, vacuum cleaning robots, and robot-like devices). | .770 | | |
| I already use a robotic assistance system in my everyday life. | .829 | | |
| I can remember a situation where this robotic arm would have been helpful for me. | | .806 | |
| I could currently use this robot as a potential support. | | .829 | |
| This robotic arm would preserve my independence. | | .647 | .608 |
| This robot would be a support for my caregivers. | | .618 | .661 |
| I would use the robotic arm for tasks that are far from my body (eg, handling or positioning of objects, and adjusting the bedspread). | | .456 | .774 |
| I would use this robot for tasks that take place near or at my body (eg, scratching, wiping off saliva, and positioning of extremities or head). | | .394 | .794 |
| The use of this robotic arm would be possible in my house. | | | .795 |
| I would like to see this robot acknowledged as an assistive medical device. | | | .718 |

Extraction Method: Principal Component Analysis.

Rotation Method: Varimax with Kaiser Normalization.

a. Rotation converged in 9 iterations.

Loadings <.3 are suppressed.
