# Supplementary figures and images for: Acceptance of Enhanced Robotic Assistance Systems in People With Amyotrophic Lateral Sclerosis–Associated Motor Impairment: Observational Online Study
Source: JMIR Rehabil Assist Technol. 2021 Dec 6;8(4):e18972. doi: 10.2196/18972 (PMC8691409; doi:10.2196/18972)

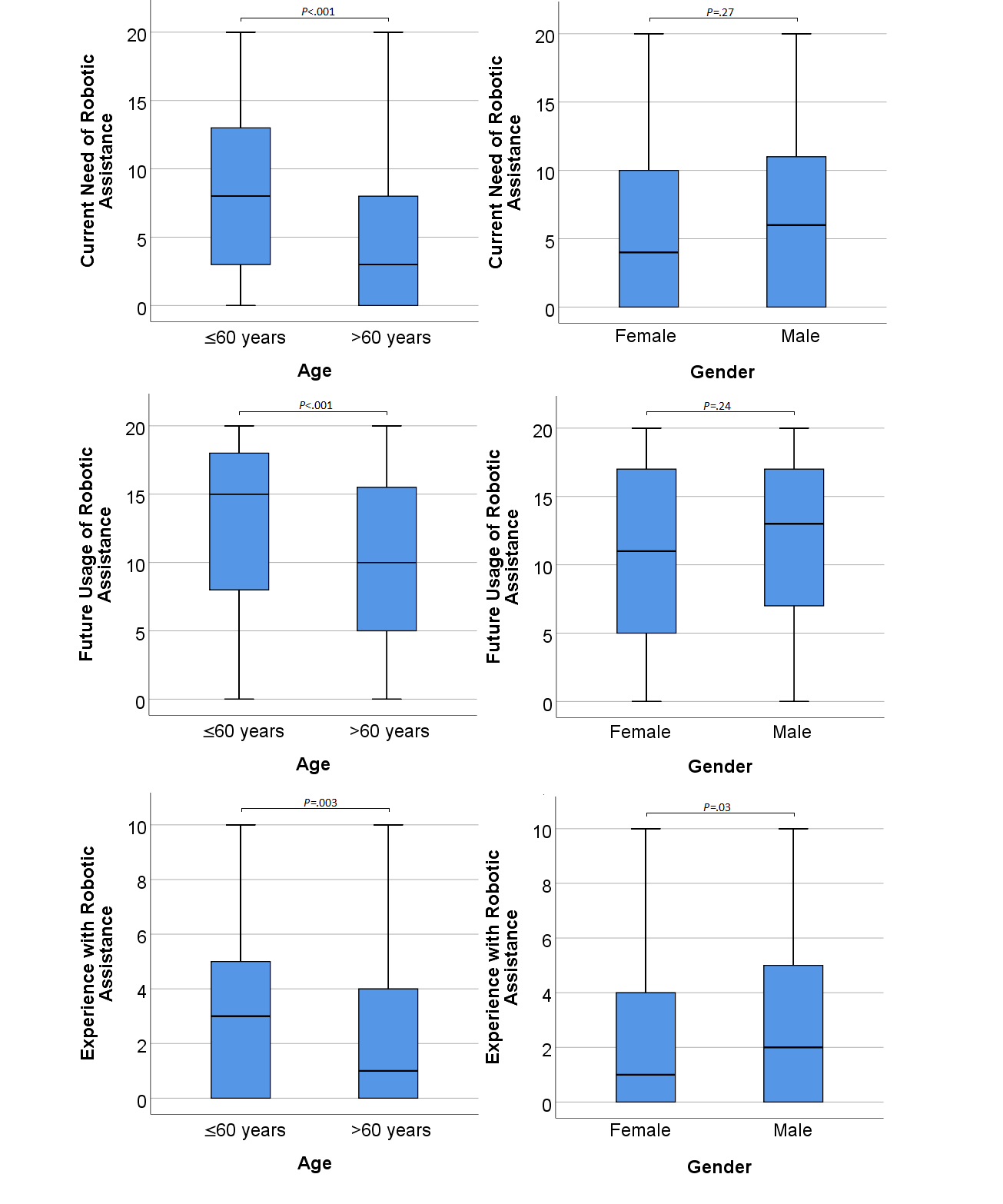

Supplement: Multimedia Appendix 4 [file rehab_v8i4e18972_app4.png]

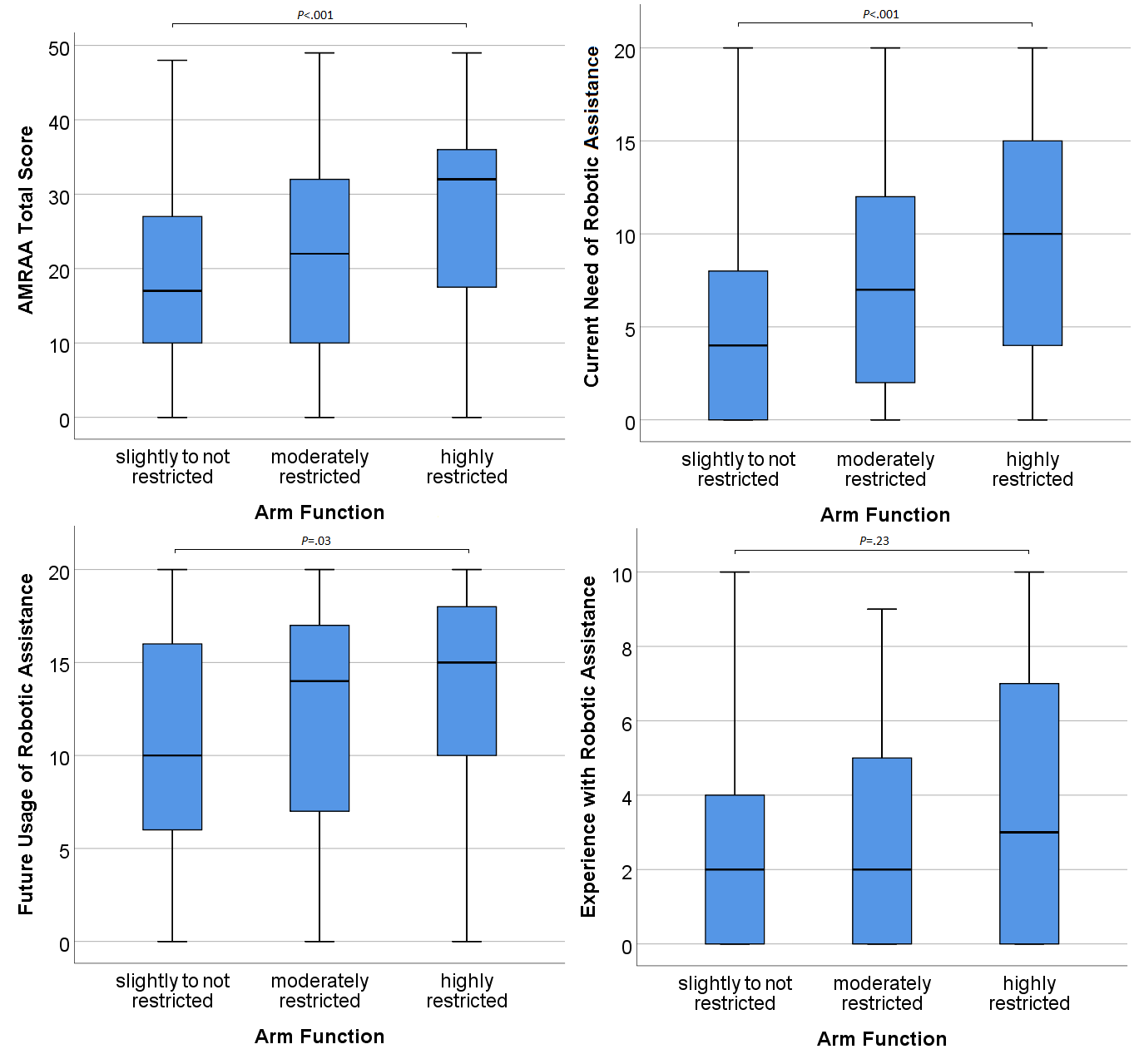

Supplement: Multimedia Appendix 5 [file rehab_v8i4e18972_app5.png]
